# Construction of a fecal immune-related protein-based biomarker panel for colorectal cancer diagnosis: a multicenter study

**DOI:** 10.3389/fimmu.2023.1126217

**Published:** 2023-05-29

**Authors:** Hao Zhang, Lugen Zuo, Jing Li, Zhijun Geng, Sitang Ge, Xue Song, Yueyue Wang, Xiaofeng Zhang, Lian Wang, Tianhao Zhao, Min Deng, Damin Chai, Qiusheng Wang, Zi Yang, Quanli Liu, Quanwei Qiu, Xuxu He, Yiqun Yang, Yuanyuan Ge, Rong Wu, Lin Zheng, Jianjun Li, Runkai Chen, Jialiang Sun, Jianguo Hu

**Affiliations:** ^1^ Department of Gastrointestinal Surgery, First Affiliated Hospital of Bengbu Medical College, Bengbu, China; ^2^ Department of Inflammatory Bowel Disease Research Center, First Affiliated Hospital of Bengbu Medical College, Bengbu, China; ^3^ Department of Clinical Laboratory, First Affiliated Hospital of Bengbu Medical College, Bengbu, China; ^4^ Department of Central Laboratory, First Affiliated Hospital of Bengbu Medical College, Bengbu, China; ^5^ Department of Gastroenterology, First Affiliated Hospital of Bengbu Medical College, Bengbu, China; ^6^ Department of Pathology, First Affiliated Hospital of Bengbu Medical College, Bengbu, China; ^7^ Department of Colorectal Surgery, Nanjing Hospital of Chinese Medicine Affiliated to Nanjing University of Chinese Medicine, Nanjing, China; ^8^ Department of General Surgery, Zhongda Hospital, Southeast University, Nanjing, China; ^9^ Department of Clinical Laboratory, The Affiliated Hospital of Medical School of Ningbo University, Ningbo, China; ^10^ Department of General Surgery, The First Medical Center, Chinese PLA General Hospital, Beijing, China; ^11^ Department of General Surgery, Chinese PLA General Hospital, Beijing, China; ^12^ Department of General Surgery, Shanghai Fengxian District Central Hospital, Shanghai, China

**Keywords:** colorectal cancer, fecal protein, immune-related protein, proteomics, multicenter study

## Abstract

**Purpose:**

To explore fecal immune-related proteins that can be used for colorectal cancer (CRC) diagnosis.

**Patients and methods:**

Three independent cohorts were used in present study. In the discovery cohort, which included 14 CRC patients and 6 healthy controls (HCs), label-free proteomics was applied to identify immune-related proteins in stool that could be used for CRC diagnosis. Exploring potential links between gut microbes and immune-related proteins by 16S rRNA sequencing. The abundance of fecal immune-associated proteins was verified by ELISA in two independent validation cohorts and a biomarker panel was constructed that could be used for CRC diagnosis. The validation cohort I included 192 CRC patients and 151 HCs from 6 different hospitals. The validation cohort II included 141 CRC patients, 82 colorectal adenoma (CRA) patients, and 87 HCs from another hospital. Finally, the expression of biomarkers in cancer tissues was verified by immunohistochemistry (IHC).

**Results:**

In the discovery study, 436 plausible fecal proteins were identified. And among 67 differential fecal proteins (|log2 fold change| > 1, P< 0.01) that could be used for CRC diagnosis, 16 immune-related proteins with diagnostic value were identified. The 16S rRNA sequencing results showed a positive correlation between immune-related proteins and the abundance of oncogenic bacteria. In the validation cohort I, a biomarker panel consisting of five fecal immune-related proteins (CAT, LTF, MMP9, RBP4, and SERPINA3) was constructed based on the least absolute shrinkage and selection operator (LASSO) and multivariate logistic regression. The biomarker panel was found to be superior to hemoglobin in the diagnosis of CRC in both validation cohort I and validation cohort II. The IHC result showed that protein expression levels of these five immune-related proteins were significantly higher in CRC tissue than in normal colorectal tissue.

**Conclusion:**

A novel biomarker panel consisting of fecal immune-related proteins can be used for the diagnosis of CRC.

## 1 Introduction

Colorectal cancer (CRC) is one of the most common malignancies, early detection and treatment are effective means to improve the prognosis of CRC (1, 2). In the clinic, assessment of hematological tumor markers is currently the main method for CRC screening, but the diagnostic sensitivity and specificity of these markers are unsatisfactory (3, 4). Although colonoscopy has significantly improved the accuracy of CRC diagnosis, it is not suitable for clinical screening due to its invasiveness and low compliance (5, 6). Thus, a noninvasive, low-cost, and high-efficacy diagnostic screening method is urgently needed.

Stool is regarded as a proxy for gut health and may provide useful information for the diagnosis of CRC. For example, the fecal occult blood test (FOBT) has been used for CRC screening for many years, although it is not very effective (7, 8). Several recent proteomic studies in Europe and North America have demonstrated the use of fecal proteins for CRC diagnosis (9, 10). However, there are still differences in the biomarkers screened in these studies, which may be due to ethnic differences and differences in dietary habits. Unfortunately, there is a lack of fecal proteomics studies from China, a high prevalence area for CRC.

In addition, alterations in stool protein composition and abundance remain unexplained. Tumor growth can damage the intestinal mucosal epithelium, leading to immune cell infiltration, which can induce intestinal barrier injury even in the early stages of CRC (11). Gut bacteria can also translocate into the tumor microenvironment (TME) and exacerbate tumor-related inflammation (12). These clues suggest that more immune cells and/or proteins may be present in the stool of CRC patients and may provide assistance in the diagnosis of CRC.

In the present study, we detected immune-related proteins in the stool of patients with CRC and healthy controls (HCs) by label-free quantitative proteomics. We also found that immune-related proteins were positively correlated with the abundance of pathogenic bacteria in CRC. Subsequently, the abundance of immune-associated proteins in feces was verified based on ELISA means in validation cohorts, and a novel biomarker panel consisting of five fecal immune-related proteins was developed for the diagnosis of CRC. In conclusion, this study provides new insights into the diagnosis and pathogenesis of CRC.

## 2 Materials and methods

### 2.1 Study design and study population

**Figure 1:**
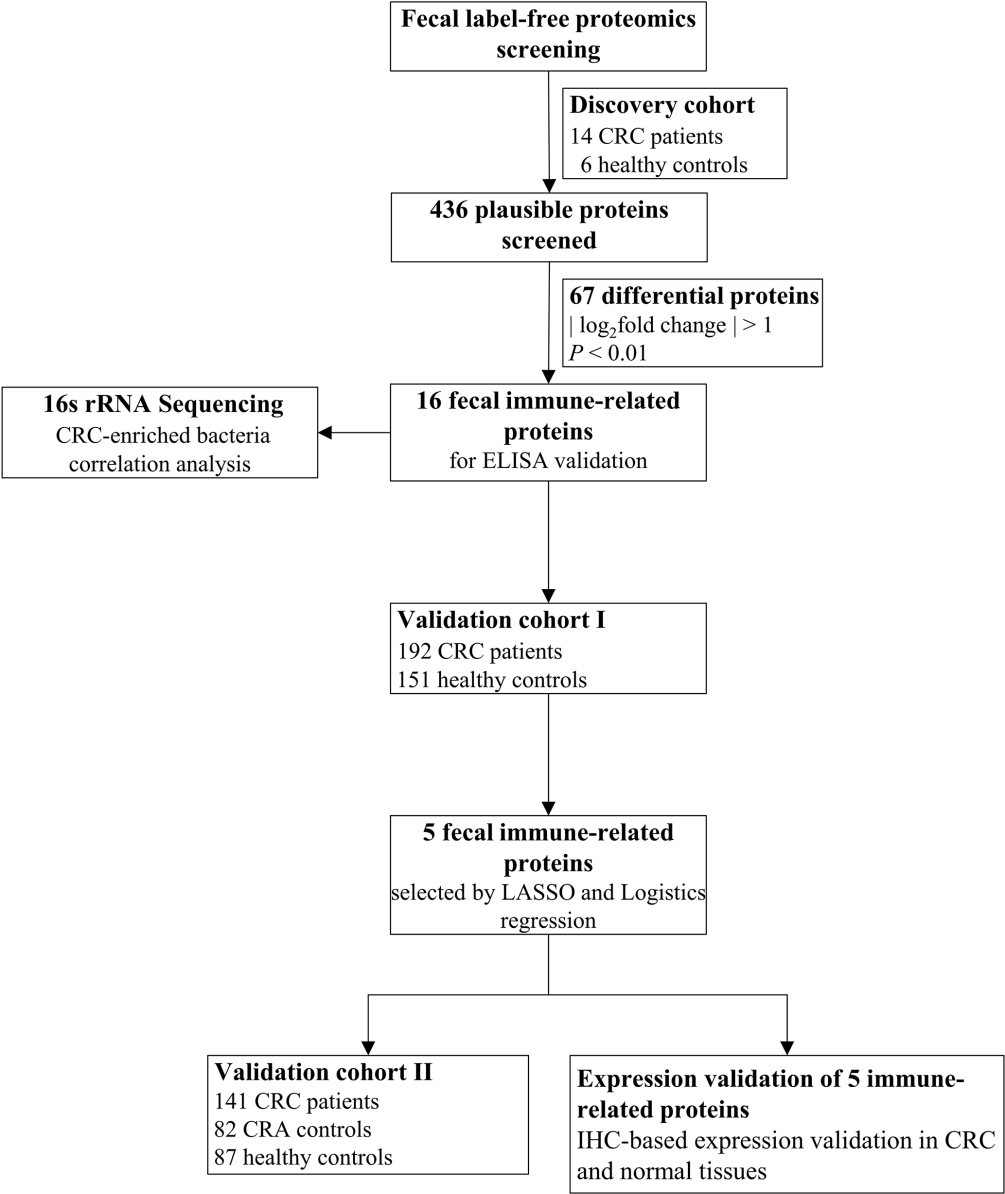
Flow diagram of the current study.


As shown in **Figure 1**, we designed a multicenter study, consisting of a discovery cohort and two validation cohorts, to identify human immune-related proteins in stool that could be used for CRC diagnosis. The discovery cohort included a case–control cohort, with 14 CRC patients and 6 HCs, and the study was performed at a single university-affiliated hospital. As previously reported (13), the validation cohort sample size (n) was calculated according to the following formula:

$$\text{n}=\frac{\text{P}}{\left(\text{S}-1\right)\ln\left(1-\frac{{\text{R}}_{\text{ cs}}^{2}}{\text{S}}\right)}$$

where the expected contraction factor (S) is calculated by the formula $\text{S}=\frac{{\text{R}}_{\text{ cs}}^{2}}{{\text{R}}_{\text{ cs}}^{2}{+\delta \max(\text{R}}_{\text{ cs}}^{2})}$ , with R^2^_cs_ = 0.2, max(R^2^_cs_) = 0.9, and δ = 0.05. Finally, validation cohort I included 343 patients from six different hospitals, with 192 CRC patients and 151 HCs. In addition, 141 CRC patients, 82 CRA controls, and 87 HCs from a single university-affiliated hospital were included in validation cohort II. All donors recruited for this study were of Han Chinese population. The study was approved by the Ethics Committee of Bengbu Medical College (approval number 2021-221). The general clinical characteristics of each group in the corresponding cohort are shown in **Supplementary Table 1**.

### 2.2 Stool sample collection

According to previous reports (9), all stool samples were collected with a standard operating procedure (SOP) collection method. All donors were instructed as to collection procedures prior to stool collection. The standard 5 mL stool collection kit was used to collect stool samples from donors without bowel preparation and under nonfasting conditions. The sample was excluded if the stool was watery in nature or if the donors had been treated with preoperative chemotherapy or radiotherapy prior to collection. The stool samples were transferred to a -80°C freezer in an ice box within 1 hour. Samples were stored at this temperature until proteomic assays were performed. All subjects provided written consent for their stool samples to be used.

### 2.3 Label-free quantitative proteomics

The frozen stool samples were removed, liquid nitrogen was added, and the samples were thoroughly ground. Then, 1 mL of extraction solution was added, and the sample was mixed well. An equal volume of phenol-Tris-HCl (pH 7.8) saturated solution was added, and the sample was mixed for 30 min at 4°C. The sample was then centrifuged at 7100 × g for 10 min. The upper layer was collected, a 5x volume of 0.1 M ammonium acetate-methanol solution was added, and the sample was stored at -20°C overnight. The solution was centrifuged at 12,000 × g for 10 min at 4°C, and the precipitate was collected. Then, 5 times the volume of methanol was added for washing, and the sample was centrifuged at 12,000 × g for 10 min. The precipitate was collected, and this step was repeated once. The previous process was repeated twice, replacing methanol with acetone. The solution was treated with SDS lysis buffer (P0013G, Beyotime Biotechnology, Shanghai, China), lysed at room temperature for 3 h, and centrifuged at 12,000 × g for 10 min. The supernatant was removed and centrifuged again at 12,000 × g for 10 min. The supernatant was the total protein sample, which was stored until the experiment was performed.

For each sample, 50 µg of protein solution was taken, mixed with DTT solution (Sangon, Shanghai, China), incubated at 55°C for 30 min, cooled to room temperature on ice, mixed with iodoacetamide and allowed to stand for 15 min at room temperature, protected from light. A 6x volume of acetone was added to precipitate the protein, and the sample was stored overnight at -20°C. The precipitated peptides were collected by centrifugation at 8000 × g for 10 min at 4°C. Then, 100 μL of TEAB2 (Sigma, USA) was added to resolubilize the precipitate, 1 mg/ml trypsin-TPCK (Sangon) was added to 1/50 of the sample mass, and the peptides were obtained after digestion at 37°C overnight. The peptides were then desalted using SOLA™ SPE 96-well plates (Thermo Fisher Scientific, USA) and analyzed on a QE mass spectrometer (Thermo Fisher Scientific) equipped with an Easyspray source (Thermo Fisher Scientific) to obtain the final LC-MS/MS raw files for subsequent analysis.

The LC-MS/MS raw files were searched for LFQ nonstandard quantitative analysis using MaxQuant (1.6.17.0). To prevent peak mismatches, the search criteria were controlled at false discovery rate (FDR)<0.01, and null values that did not meet the analytical criteria were excluded. Proteins without missing values in more than half of the samples were retained as plausible proteins, and the missing values were filled with the mean of the same group. Ultimately, the plausible proteins were median normalized and log-transformed. Differences between the two groups were assessed based on the fold change in protein abundance, and p values were calculated by a two-tailed t test. The criteria for differentially expressed proteins were fold change >1.2 and P<0.05.

### 2.4 ELISA-based protein validation

After the stool sample was weighed, fecal protein was extracted using a fecal protein lysis solution based on the Tris-HCl method (containing 20 mM Tris-HCl, 1% Triton X-100, and protease inhibitor; lysis solution, pH 7.5), and the detailed procedure was as follows: first, the stool samples were thawed, then one gram of stool was weighed and removed from the samples, and four milliliters of fecal protein lysis solution was added. The sample was then thoroughly shaken for 30 seconds with a vortexer, allowed to stand on ice for 5 min, thoroughly shaken again for 30 seconds with a vortexer and allowed to stand on ice for 20 min. The rest of the solution was first centrifuged at low speed (2500 rpm, 5 min, 4°C), after which the lower layer was discarded and the supernatant was aspirated, followed by ultracentrifugation (12000 rpm, 30 min, 4°C). The lower layer was discarded, and the supernatant was aspirated into 1.5 ml EP tubes. After quantifying the total protein concentration by the BCA method, the protein levels of 16 biomarkers (A2M, APOD, C3, CAT, CYBB, GPI, IGHG2, IGKV1-5, LTF, MMP9, ORM1, PGLYRP1, RBP4, S100A6, SERPINA3, and SERPIND1) in the stool sample were measured according to the ELISA kit instructions. Information on all ELISA kits used in this study can be found in **Supplementary File 1**.

### 2.5 Immune gene list

ImmPort (https://www.immport.org/) is a public database funded by the NIH, NIAID and DAIT that shares data provided by NIH-funded projects, other research organizations and individual scientists (14, 15). The list of immune genes/proteins in the ImmPort database was obtained based on functions and Gene Ontology terms. In total, 2483 immune genes/proteins were obtained from the ImmPort database.

### 2.6 Immunohistochemistry

The levels of five proteins in the biomarker panel were determined by immunohistochemical analysis in colorectal tumor tissue and adjacent normal tissue, as we previously described (16, 17). Tumor tissue from CRC patients undergoing surgery in the discovery cohort and adjacent normal tissue more than 5 cm away from the tumor lesion were collected. Paraffin tissue blocks approximately 2*2 cm in size were prepared after soaking in formalin solution. Paraffin blocks were cut into 4 μm sections, removed, treated with antigen repair solution, blocked with goat serum, and incubated with primary antibodies overnight at 4°C. The antibodies used included anti-CAT (1:1000, ab76024, Abcam, Cambridge, MA, USA), anti-LTF (1:2500, ab262902, Abcam), anti-MMP9 (1:800, ab137867, Abcam), anti-RBP4 (1:500, ab133559, Abcam) and anti-SERPINA3 (1:500, ab205198, Abcam). The samples were then incubated with a 1:300 dilution of goat anti-rabbit immunoglobulin G (IgG) antibody (A0286, Beyotime Biotechnology) in PBS for 1 h at room temperature. Subsequently, the samples were stained with 3,3’-diaminobenzidine (DAB kit, P0203, Beyoncé), dehydrated and sealed. Negative control sections were prepared in the same manner as adjacent normal tissues except that no primary antibody was added. Five fields of view from each tissue section were randomly selected for quantification of integrated optical density values of immune-related proteins.

### 2.7 Statistical analysis

Data analysis was performed using GraphPad 9.0.0 (San Diego, CA, USA) and R 4.2.0. Comparisons between baseline characteristics were made using chi-squared tests. Comparisons between paired data were made using paired t-tests. Correlations between biomarkers, including proteins and microbes, were analyzed with the Spearman rank correlation test. The Wilcoxon test was used for comparisons between two groups. The least absolute shrinkage and selection operator (LASSO) and multivariate logistic regression analysis were employed by the R package “glmnet.” The receiver operating characteristic (ROC) curve analysis with 10-fold cross-validation and calculation of the area under the curve (AUC) were performed with the R “pROC” package. The DeLong test was used to compare the differences between AUC values. Calibration curve and decision curve analyses were performed with the R packages “rms” and “ggDCA.” The results were considered statistically significant at *P<* 0.05.

## 3 Results

### 3.1 Fecal immune-related proteins distinguished CRC patients from HCs in the discovery cohort

**Figure 2:**
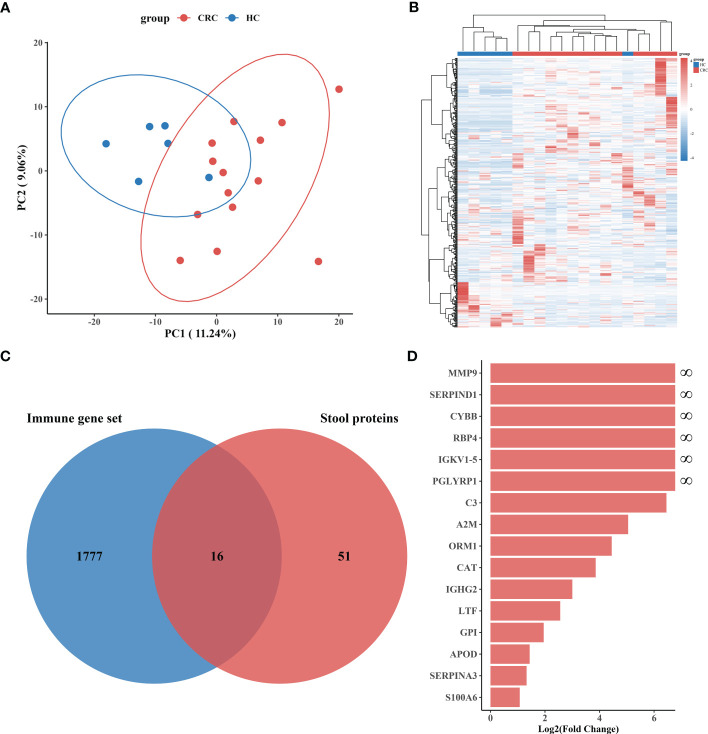
Screening and identification of immune-related proteins in stool. **(A)** Principal component analysis (PCA) of discovery cohort I stool protein profile data. The red dots represent the CRC group (n = 14), and the blue dots represent the HC group (n = 6). Each dot represents a sample, and the distance between the dots represents the degree of variation between samples. **(B)** Cluster heatmap of all 436 plausible stool proteins. **(C)** Venn diagram showing immune-related proteins as common biomarkers in differentially expressed stool proteins and immune genes. **(D)** Log_2_(Fold Change) bar plots demonstrate the degree of difference between the CRC and HC samples for 16 immune-related proteins, all of which were present at higher levels in the CRC group.

Proteomics data were subjected to rigorous quality control, after which 436 plausible human proteins were identified from the fecal samples of the discovery cohort. We further checked the abundance of specific proteins in the stool samples, and the results showed that the hemoglobin alpha chain (HBA1) levels were strongly associated with the beta chain (HBB) levels (**Supplementary Figure 1A**). Two components of calprotectin (S100A8 and S100A9) showed similar results (**Supplementary Figure S1B**). Unsupervised principal component analysis (PCA) was performed, and an unsupervised clustering heatmap was generated for all stool proteins, revealing significant differences in samples from patients with CRC versus HCs (**Figures 2A, B**). We then performed a differential analysis of protein abundance to obtain stool proteins that could be used to distinguish between the CRC group and the HC group. The results revealed that 97 proteins (fold change > 1.2, *P<* 0.05) could be utilized to differentiate CRC patients from HCs, with 72 of these proteins being more abundant in stool samples from CRC patients (**Supplementary Figure 2**). To further improve the specificity of differentially expressed protein identification, we employed more stringent selection thresholds (|log_2_-fold change| > 1, *P<* 0.01) and ultimately identified 67 differential proteins, 55 of which were more abundant in the CRC group samples (**Supplementary Figure 3**).

The occurrence and progression of CRC are accompanied by complex local intestinal immune activities, and immune-related proteins with potential diagnostic value may appear in the stool.

The 67 differentially expressed fecal proteins identified in Cohort I were matched to immune genes in the ImmPort database, and we ultimately identified 16 immune-related proteins for CRC diagnosis (**Figure 2C**). All of these immune-related proteins were found in CRC patients or in high abundance (**Figure 2D**). Among the 16 immune-related proteins, 6 proteins (MMP9, CYBB, RBP4, IGKV1-5, SERPIND1 and PGLYRP1) were only present in the stool of CRC patients, while the remaining 10 proteins (C3, A2M, ORM1, CAT, IGHG2, LTF, GPI, APOD, SERPINA3, and S100A6) were present at elevated levels in the stool of CRC patients (**Supplementary Table 2**).

These results confirm that it is possible to distinguish CRC patients from HCs by immune-related proteins in the stool.

### 3.2 Fecal immune-related protein validation and construction of a biomarker panel in validation cohort I

**Figure 3:**
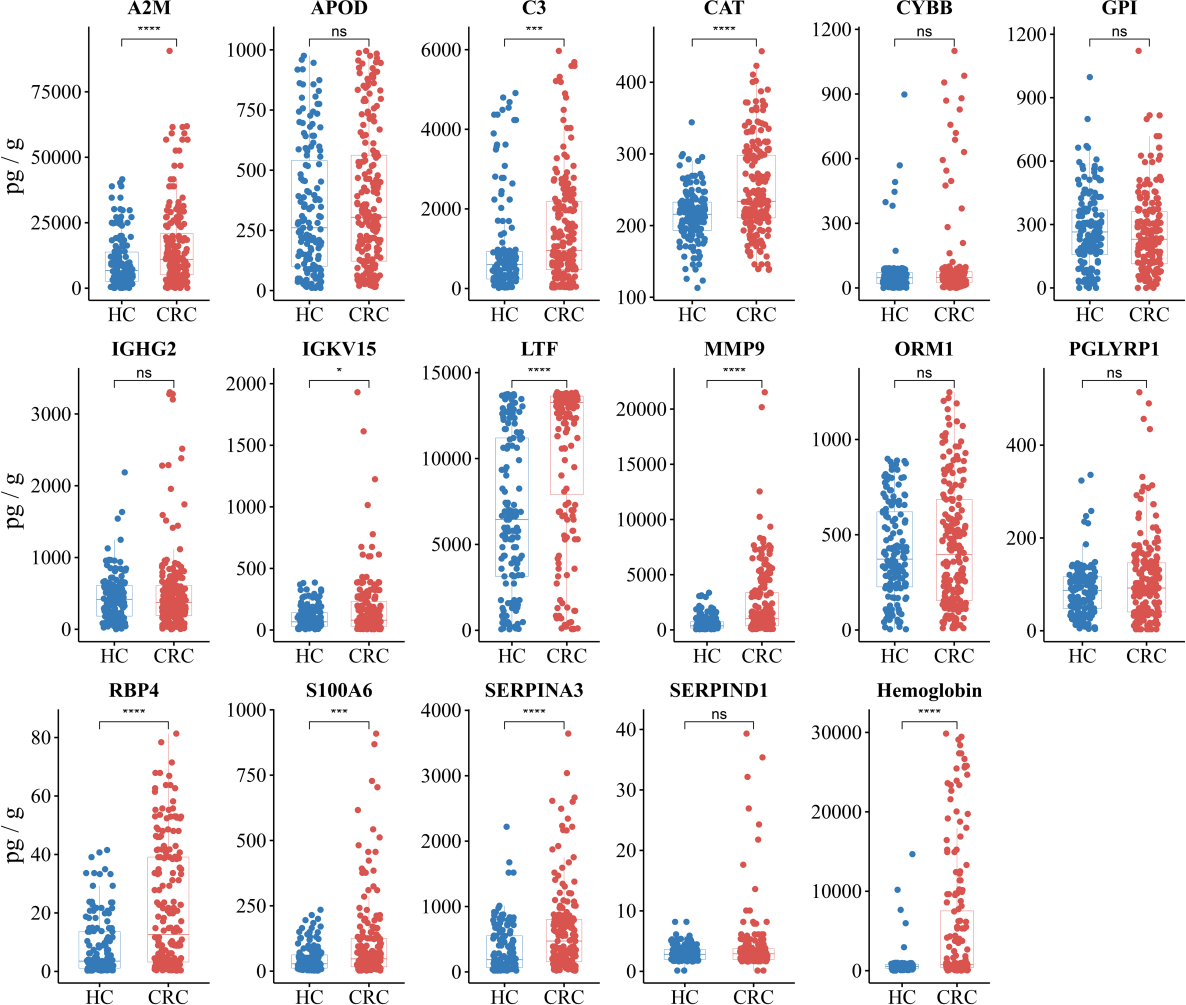
Protein abundance assays and differential analysis of 16 immune-related proteins and hemoglobin were performed in validation cohort I. Validation cohort I (n = 343) included 192 CRC patients (red) and 151 HCs (blue), with each dot representing one sample. The unpaired Wilcoxon rank test was used to analyze differences in protein abundance. ^*^ *P*< 0.05, ^***^ *P*< 0.001, ^****^ *P*< 0.0001, ns, not significant.

For the purpose of clinical application, the 16 identified immune-related proteins in stool were tested by ELISA in the independent validation cohorts. The first independent validation study included 192 CRC patients and 151 HCs. To evaluate the efficacy of the protein panel more accurately, we chose hemoglobin, which is commonly used in clinical practice, as a control biomarker. The ELISA results revealed that the levels of nine immune-related proteins (A2M, C3, CAT, IGKV1-5, LTF, MMP9, RBP4, S100A6, and SERPINA3) were significantly higher in the CRC group than in the HC group (*P<* 0.05). The levels of the other seven proteins (APOD, CYBB, GPI, IGHG2, ORM1, PGLYRP1, and SERPIND1) were not significantly different between the two groups (*P* > 0.05), although the abundance of these proteins was higher in the CRC group (**Figure 3**).

**Figure 4:**
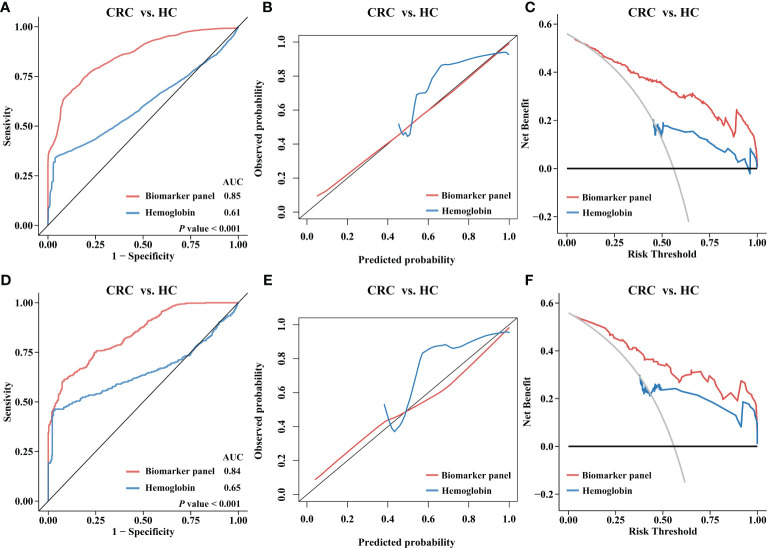
Diagnostic performance of the biomarker panel in validation cohort I and results compared to those for hemoglobin. Validation cohort I (n = 343) was randomly divided into a training set (n = 241) and a testing set (n = 102). The biomarker panel included CAT, LTF, MMP9, RBP4, and SERPINA3. **(A)** Receiver operating characteristic (ROC) curve analysis of the biomarker panel and hemoglobin in the training set. The area under the curve (AUC) was compared using the DeLong test. **(B)** Calibration curve analysis of the biomarker panel and hemoglobin in the training set. The closer the curve is to the reference line (black), the more accurate the prediction. **(C)** Decision curve analysis (DCA) for the biomarker panel and hemoglobin in the training set. The larger the area under the curve is, the greater the clinical benefit that can be expected. **(D)** ROC curves, **(E)** calibration curve, **(F)** DCA curve for the biomarker panel and hemoglobin in the testing set.

To construct the biomarker panel, the validation cohort was randomly split into a training set and a testing set. In the training set, LASSO regression and multivariate logistic regression were performed to identify stool immune-related proteins that could be used to detect CRC (**Supplementary Figure 4**, **Supplementary Table 3**). Finally, a biomarker panel of five proteins (CAT, LTF, MMP9, RBP4, and SERPINA3) was identified. ROC curve analysis showed that the biomarker panel had a significantly higher adjusted AUC value than hemoglobin (*P*< 0.05) (**Figure 4A**). The calibration curve also showed that the predictions obtained with the biomarker panel compared to those obtained based on the use of hemoglobin were closer to the true results (**Figure 4B**). DCA results showed that the use of our panel of biomarkers based on fecal immune-related proteins resulted in significantly higher net gains than the use of hemoglobin (**Figure 4C**). We also observed that the biomarker panel outperformed hemoglobin in the testing set at identifying CRC patients (**Figures 4D–F**).

### 3.3 Diagnostic capability of the biomarker panel in validation cohort II

**Figure 5:**
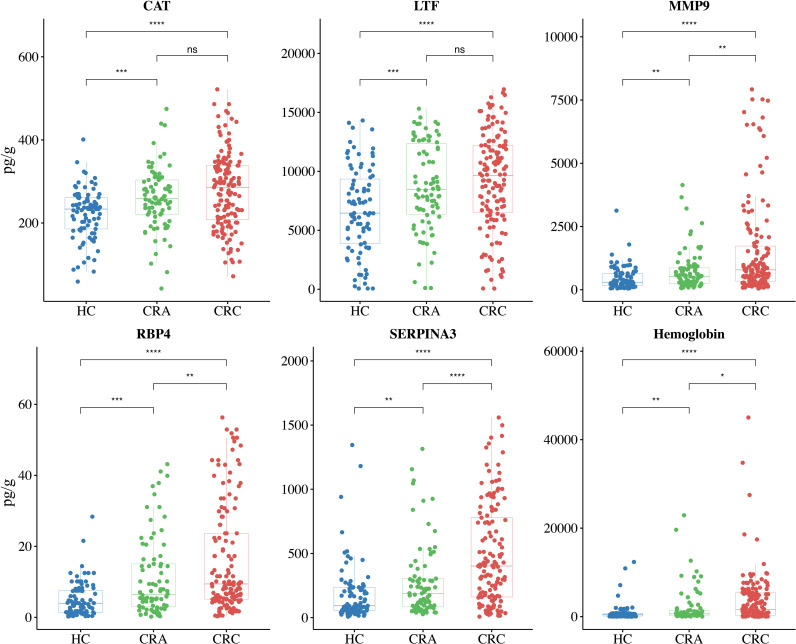
Protein abundance assays and differential analysis of the biomarker panel and hemoglobin were performed in Validation cohort II. The biomarker panel included CAT, LTF, MMP9, RBP4, and SERPINA3. Validation cohort II (n = 310) included 141 CRC patients (red), 82 CRA patients (green), and 87 HCs (blue), with each dot representing one sample. The unpaired Wilcoxon rank test was used to analyze differences in protein abundance. ^*^ *P*< 0.05, ^**^ *P*< 0.01, ^***^ *P*< 0.001, ^****^ *P*< 0.0001, ns, not significant.

**Figure 6:**
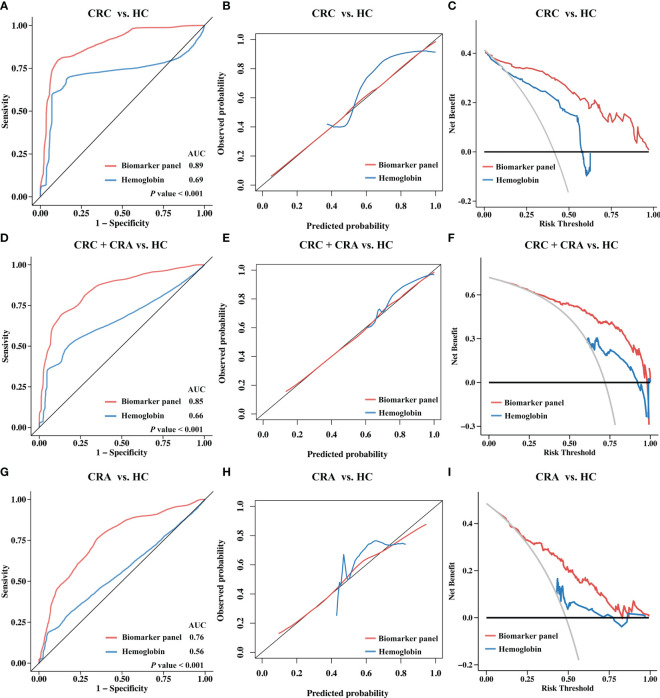
Diagnostic performance of the biomarker panel in Validation Cohort II and results compared to hemoglobin. Validation cohort II (n = 310) included 141 CRC patients, 82 CRA patients, and 87 HCs. The biomarker panel included CAT, LTF, MMP9, RBP4, and SERPINA3. **(A)** Receiver operating characteristic (ROC) curve analysis of biomarkers between the CRC and HC groups. The area under the curve (AUC) was compared using the DeLong test. **(B)** Calibration curve analysis of the biomarker panel between the CRC and HC groups. The closer the curve is to the reference line (black), the more accurate the prediction. **(C)** Decision curve analysis (DCA) for the biomarker panel between the CRC and HC groups. The larger the area under the curve is, the greater the clinical benefit that can be expected. **(D)** ROC curves, **(E)** calibration curve, **(F)** DCA curve for the biomarker panel and hemoglobin between the CRC+CRA and HC groups. **(G)** ROC curves, **(H)** calibration curve, **(I)** DCA curve for the biomarker panel and hemoglobin between the CRA and HC groups.

To further assess the efficacy of the biomarker panel in identifying CRC, we again validated the results by ELISA in a new independent cohort. Stool samples for this cohort were obtained from another hospital, and the cohort included 141 CRC patients, 82 CRA patients, and 87 HCs. As shown in **Figure 5**, the levels of CAT, LTF, MMP9, RBP4, and SERPINA3 were significantly higher in the stools of CRC patients than in the stools of HCs. ELISA results also revealed that CRA patients had higher levels of CAT, LTF, RBP4, and SERPINA3 than HCs, while the MMP9 levels were not different. ROC curve analysis revealed that the biomarker panel had an AUC value of 0.89 after 10-fold cross-validation, while hemoglobin had an AUC value of 0.69, showing that the biomarker panel still maintained a good ability to discriminate between CRC patients and HCs (**Figure 6A**). The results of the calibration curves and DCA also showed that the biomarker panel was superior to hemoglobin in terms of calibration and clinical benefit (**Figures 6B, C**).

We then compared the ability of the biomarker panel to hemoglobin for colorectal neoplasms detection. The results showed that after cross-validation, the biomarker panel (AUC=0.85) was significantly more effective(*P*<0.001) than hemoglobin (AUC=0.66) in distinguishing colorectal neoplasms (**Figure 6D**). The results of the calibration curve and DCA also showed that the biomarker panel showed better calibration and clinical benefit than hemoglobin in identifying colorectal neoplasms (**Figures 6E, F**). We also compared the capability of the biomarker panel and hemoglobin to detect benign colorectal adenomas. The results showed that the biomarker panel also performed better than hemoglobin in terms of diagnostic performance, including discriminatory capability (AUC=0.76 *vs*. AUC=0.56, P<0.001) (**Figure 6G**), calibration (**Figure 6H**) and clinical benefit (**Figure 6I**).

These results suggest that this biomarker panel performs better than hemoglobin in identifying colorectal tumors, both benign and malignant, indicating that it has potential clinical applications.

### 3.4 Validation of immune-related protein expression in CRC tissue

**Figure 7:**
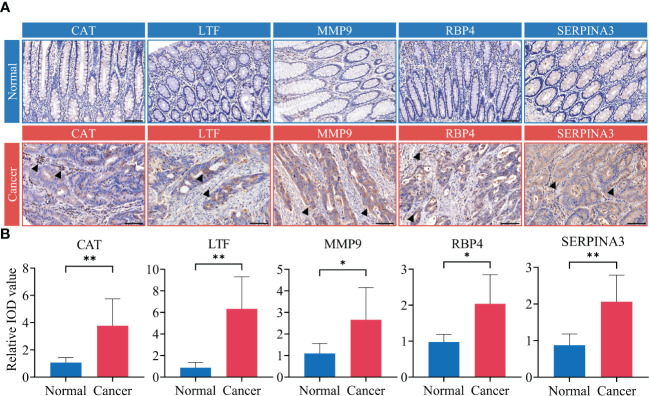
Validation of biomarker panel expression in tumor tissue. Tumor tissue samples from CRC patients in discovery cohort (n=14). The biomarker panel included CAT, LTF, MMP9, RBP4, and SERPINA3. **(A)** Immunohistochemistry (IHC)-based detection of biomarker panel expression in CRC tumor tissue and adjacent normal tissue. Black arrows indicate where the biomarker is significantly overexpressed. Scale bar represents 100 μm. **(B)** Differential analysis of IHC integrated optical density (IOD) values. Analyses were performed using paired t-test. ^*^ *P*< 0.05, ^**^ *P*< 0.01.

To further clarify the source, we confirmed the expression levels and cellular localization of five immune-related proteins by IHC in tumor tissue and adjacent normal tissue from CRC patients in the discovery cohort. The results showed that these immune-related proteins, including CAT, LTF, MMP9, RBP4 and SERPINA3, were generally undetectable or expressed at low levels in colorectal normal tissues but were significantly more highly expressed in CRC tissues (**Figure 7A**). We found that CAT and LTF were highly expressed in immune cells. In contrast, we found that MMP9 was only expressed in the cytoplasm of cancer cells but was expressed at low levels in immune cells. RBP4 and SERPINA3 were expressed in both the cancer cell cytoplasm and the immune cell cytoplasm. The results also showed statistically significant differences in the expression of immune-related proteins between CRC tissue and normal colorectal epithelial tissue (**Figure 7B**).

Based on these data, we confirmed that the level of immune-related proteins in stool remained consistent with the expression level within the tumor tissue.

## 4 Discussion

In the present study, we identified fecal tumor immune-related proteins as biomarkers for the detection of CRC based on the Han Chinese population. A total of 436 human proteins in the stool of enrolled CRC patients and HCs were identified by a label-free quantitative proteomics approach in the identification study. By setting restrictive conditions, 67 fecal proteins were found to be significantly differentially expressed between CRC patients and HCs. Among them, the levels of 16 tumor immune-related proteins were elevated in the feces of CRC patients and had potential diagnostic value. Through two independent validation cohorts, we finally identified five fecal tumor immune-related proteins (CAT, LTF, MMP9, RBP4, and SERPINA3) as well as a biomarker panel that had definite diagnostic value for CRC. In addition, we confirmed that these five proteins were highly expressed in tumor tissues, and their expression levels were positively correlated with the abundance of CRC-enriched bacteria.

High-throughput proteomics-based platforms have become one of the most powerful tools for screening cancer-specific biomarkers (18), and fecal protein analysis might be the most appropriate approach for large-scale screening for CRC. Although a few studies have reported the application of fecal protein biomarkers in CRC screening, the results of these studies appear to be, without exception, limited by region and ethnicity (9, 19). More importantly, the differentially expressed fecal proteins screened by these studies for CRC diagnosis were inconsistent. This limitation manifested specifically as significant differences in protein species and abundance in the results of studies from different regions, which may be related to ethnicity and region. In this study, we identified 67 differentially expressed proteins, but there were still some differences between these differentially expressed proteins and those in previous reports. This demonstrates the necessity of performing appropriate clinical studies in different regions to facilitate the application of regional CRC screening.

The current study highlighted the value of fecal tumor immune-related proteins in the diagnosis of CRC. Immune-related genes have been demonstrated to be useful in the diagnosis of CRC and even to be more sensitive than tumor-related genes in reflecting certain pathological processes of CRC, such as distant metastasis (20, 21). In fact, in the early course of CRC, tumor growth can injure the intestinal mucosal tissue and trigger an inflammatory/immune response that triggers the excretion of exfoliated cells/proteins in the feces. In addition, tumor immune-related proteins secreted by immune cells or tumor cells are involved in various tumor biological behaviors in CRC, including tumor metastasis, angiogenesis, extracellular matrix remodeling, and epithelial-mesenchymal transition (EMT). It is possible that these complex tumor behaviors are reflected in fecal proteins, thereby providing diagnostic clues regarding the presence of CRC. In the present study, we identified 16 immune-related proteins among the 67 significantly altered proteins in the stool samples of CRC patients. The elevated concentrations of most of the immune-related proteins in the stool of CRC patients were validated by ELISA. In addition, the biomarker panel consisting of the immune-related proteins CAT, LTF, MMP9, RBP4, and SERPINA3 showed superior discrimination ability over hemoglobin in independent validation cohorts.

Catalase (CAT) plays a key role in the protection of cells from the cytotoxic effects mediated by reactive oxygen species (ROS) (22). During tumor development, tumor cells often reduce cell death and DNA damage caused by high levels of ROS by inducing the expression of CAT (23, 24). CAT activity is increased in colon tumors due to the ability of this enzyme to reduce ROS levels in tumor cells, but this also activates leukocytes in the tumor microenvironment (25). Conversely, silencing of CAT leads to maintenance of colon cancer cells in a senescent state, thereby inhibiting tumor progression (26). In addition, CAT inactivation has been shown to contribute to the enhancement of the macrophage defense responses (27). Our data also confirm that CAT is expressed at higher levels in both stool and tumor tissue of CRC patients than in normal samples. We found that CAT is mainly expressed within the cytoplasm of tumor cells and immune cells. These results suggest that CAT may be involved in not only tumor growth but also immune activation in the tumor microenvironment.

Lactoferrin (LTF), a member of the iron transport protein (transferrin) family, has recently been recognized as a multifunctional protein with anticancer and immunomodulatory properties (28, 29). The anticancer activity of LTF can be attributed to electrostatic binding to acidic molecules highly expressed on the surface of cancer cells through its cationic N-terminal region (30). In addition, previous reports have suggested that LTF exerts anticancer activity in CRC through modulation of the host immune system (31). Several studies suggest that this activation of immunity occurs *via* LTF promoting the activation of immune cell subtypes such as leukocytes, natural killer cells, and cytotoxic T cells in the tumor microenvironment (32). We also found by IHC that LTF expression was abnormally elevated in immune cells in the CRC tumor microenvironment. Consistent with previous reports (33), we found a significantly higher abundance of LTF in the stool of CRC patients and confirmed its applicability in the diagnosis of CRC.

Matrix metalloproteinase-9 (MMP-9) is the major enzyme responsible for the degradation of type IV collagen, a major component of the basement membrane (34). This function of MMP-9 is the basis of CRC development, progression, and metastasis (35). An increasing number of studies have shown that MMP9 is associated with tumor immunity, including in T-cell depletion and immune checkpoint suppression, leading to the immune escape of tumor cells (36). In contrast, anti-MMP-9 treatment increased the expression of T-cell-associated stimulatory factors, which significantly enhanced T-cell-mediated cytotoxicity and thereby inhibited tumor progression (37). Our data show that MMP-9 is highly expressed/abundant in the tumor tissue and stool of CRC patients and can be used for CRC diagnosis, which is consistent with several previous studies that have highlighted the diagnostic ability of MMP-9 (38, 39).

Retinol binding protein 4 (RBP4) is the major carrier of retinol and is involved in vitamin A metabolism (40). There is increasing evidence that RBP4 is associated with cancer development; for example, high levels of RBP4 promote the migration and proliferation of ovarian cancer cells through stimulation of MMP2 and MMP9 expression (41). In colon cancer, RBP4 maintains the stemness of colon cancer stem cells by promoting the phosphorylation of STAT3, which affects colon cancer progression (42). The results of several microarray- or transcriptomic-based studies have shown that RBP4 is associated with the level of immune infiltration in tumor tissue (43, 44). In addition, several studies have suggested that high serum levels of rbp4 are associated with the development of colon adenomas (45). Our results showed that the expression/abundance of RBP4 was higher in tissue/stool samples from CRC patients than in normal controls, which is consistent with previous reports and confirms that RBP4 can be used for CRC diagnosis.

Serine protease inhibitor A family member 3 (SERPINA3) acts primarily as a protease inhibitor and plays an important role in the regulation of cellular processes such as oxidative stress, fibrosis, angiogenesis, inflammatory response and apoptosis (46, 47) In diabetic nephropathy, immune-related SERPINA3 inhibits mast cell proliferation and activation by downregulating chymase activity, thereby alleviating disease progression (48). During tumor progression, STAT3-dependent SERPINA3 enhances tumor cell invasion, migration, and angiogenesis (46, 49). On this basis, some oncology studies have suggested that serpina3 is associated with T-cell activation and proliferation (50, 51). In colon cancer, silencing SERPINA3 expression reduced the metastatic potential associated with colon cancer by reducing the expression of MMP2 and MMP9 (52). Previous studies have confirmed the diagnostic value of serum SERPINA3 in identifying the process of CRC development and progression (53). Consistent with previous studies, our data also showed that SERPINA3 expression was abnormally elevated in both tumor tissue and stool samples from CRC patients, indicating its diagnostic ability for CRC.

The function of these immune-related proteins in tumor immunity furnishes a theoretical foundation that buttresses the implementation of the biomarker panel devised in this study for CRC diagnosis. Notably, directing attention to the immune-related proteins constitutes a distinctive perspective of this study in contrast to previous investigations conducted in Europe and North America. In previous European research (10), 29 fecal proteins were screened for differential expression and a biomarker panel consisting of four proteins (C3, LTF, HBA1, and HP) was constructed using logistic regression. Despite noting abnormal elevations of CAT and RBP4 in CRC feces in this study, only LTF among the immune-related proteins was incorporated into the diagnostic biomarker panel. The European study also identified abnormally elevated levels of various proteins in the SERPIN family in CRC stools, however, the diagnostic capability of SERPINA3 was not validated. North American research solely presented a list of fecal proteins with diagnostic potential, yet no biomarker panel was established (9). During the North American study, only MMP9 was identified as a diagnostic marker, and although CAT and LTF were discovered to be abnormally elevated in CRC stool samples, the diagnostic validity of the remaining four immune-related proteins (CAT, LTF, RBP4, SERPINA3) has not been established. The dissimilarities observed between the outcomes of this study and those from Europe or North America are likely attributed to variances in genetic background related to different racial populations as well as variations in dietary habits.

We also attempted to analyze whether the immune-related proteins identified are associated with gut bacterial disorders in CRC, which may explain their abnormal abundance in the stool (**Supplementary Figure 5A-C**). Ectopic pathogenic bacteria have the ability to activate host immune cells and induce tumor-exogenous inflammation, ultimately leading to cellular mutation and cancer (54). Thus, an increase in the abundance of pathogenic bacteria may be a factor underlying the detection of immune-related proteins in the stool. In our results, two microbial genera, *Alistipes* and *Fusobacterium*, were enriched in the CRC group with the highest LDA scores (**Supplementary Figures 6**, **5D**).

*Alistipes* species are anaerobic bacteria found mainly in the gastrointestinal tract, and due to their unique method of fermenting amino acids, putrefaction*, Alistipes* species play a crucial role in various diseases (55–57). Previous studies have suggested that the abundance of the genus *Alistipes* and TLR4/TNF production are positively correlated (58). In an inflammatory environment lacking Lipocalin 2, *Alistipes* species grow rapidly and promote the development of inflammation and tumor formation (59). Recent research suggests that members of the genus *Fusobacterium* accelerate the transition from an inflammatory state to malignancy, particularly in colorectal cancer (60, 61). Both *in vitro* and *in vivo* experiments have confirmed that *Fusobacterium* species promote the growth of colorectal cancer cells (62–64). The mechanisms underlying the action of *Fusobacterium* could range from increasing tumor cell adherence and invasion to influencing the host immune response and activating the Toll-like receptor 4 pathway (61). These previous findings suggest that *Alistipes* and *Fusobacterium* are genera of pathogenic bacteria that promote the development of CRC and have the potential to serve as diagnostic biomarkers for CRC, which is consistent with our study. We confirmed by Spearman correlation analysis that the abundances of *Alistipes* and *Fusobacterium* were positively correlated with the abundances of tumor immune-related proteins, which is consistent with the trends for other CRC-enriched microorganisms (**Supplementary Figure 5E**). Therefore, the interaction between these bacteria and host immunity may be one of the main reasons for the presence of immune proteins in the stool. This interaction further suggests the possibility that aberrant microorganisms and immune-related proteins constitute a combination of diagnostic biomarkers.

There are some limitations to this study. Because of the relatively small number of patients included in the discovery cohort of this study, we cannot exclude the possibility that some of the more important stool proteins were not identified. Differences in diet may also have had a partial effect on the proteomic results and microbial composition. Unfortunately, we were not able to maintain the same dietary requirements for all providers, so we cannot exclude the possibility that areas with different dietary habits produced results different from those of the present study. As our study included only the Han Chinese population, further clinical studies conducted in more regions and with larger sample sizes will be necessary to validate our results and assess the generalizability of our findings to other populations. This study only explored the correlation between gut bacteria and immune-related proteins through 16S rRNA sequencing data and proteomics data, and more in-depth experiments are needed to explore the detailed mechanisms.

As the first clinical study of the stool proteome in the Han Chinese population, this study established a novel biomarker panel of fecal immune-related proteins (CAT, LTF, MMP9, RBP4, and SERPINA3) for CRC screening. The potential interaction relationship between abnormal bacteria and immune-related proteins provided new theoretical support for the origin of the proteins. Finally, this study might provide a novel perspective on the diagnostic and mechanistic study of CRC.

## Data availability statement

The proteomics data and sequencing data generated and analyzed in this study are available as follows: iProX online database (https://www.iprox.cn/) with accession number IPX0005614000, and NCBI online database (https://www.ncbi.nlm.nih.gov/) with accession number PRJNA911939. Other types of data used to support the findings of this study are available from the corresponding author upon request.

## Ethics statement

The studies involving human participants were reviewed and approved by the Ethics Committee of Bengbu Medical College. The patients/participants provided their written informed consent to participate in this study.

## Author contributions

HZ, LuZ, JinL and JH contributed to the study concept and design. HZ, LuZ, SG, MD, QW, QQ, XH, TZ, YG, RW, LiZ, JiaL, RC and JS contributed to the clinical data collected and assessed the data. JinL, ZG, YW, LW, TZ, QL, XZ, XS, DC and ZY contributed to the acquisition of the laboratory data. HZ and LuZ contributed to the data analysis and data interpretation. HZ drafted the manuscript, LuZ, JinL and JH critically revised the manuscript. JH provided research fundings, supervised the study and coordinated the research. All authors contributed to the article and approved the submitted version.
