# Supplementary material for: Construction of a fecal immune-related protein-based biomarker panel for colorectal cancer diagnosis: a multicenter study
Source: Front Immunol. 2023 May 29;14:1126217. doi: 10.3389/fimmu.2023.1126217 (PMC10258350; doi:10.3389/fimmu.2023.1126217)
Supplement: Supplementary file 1 [file Image_1.pdf]

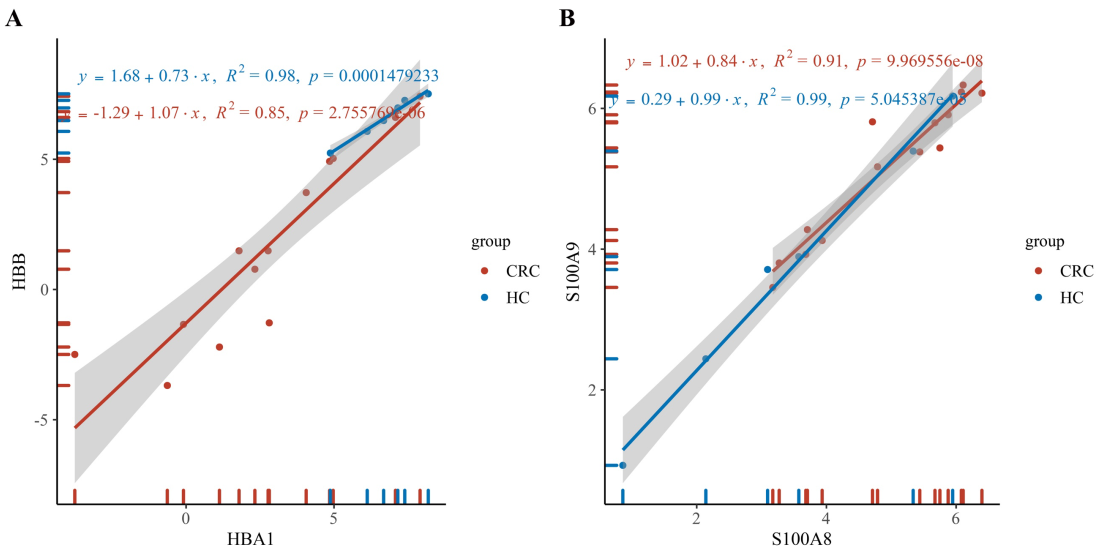


**Supplementary Figure 1. Stool proteomic characteristics of CRC patients in Discovery Cohort.** Discovery cohort included 14 CRC patients and 6 HCs. (A) Spearman correlation analysis of the hemoglobin components HBA1 and HBB. Each dot represents a sample. (B) Spearman correlation analysis of S100A8 and S100A9. Each dot represents a sample.
