# Supplementary material for: Construction of a fecal immune-related protein-based biomarker panel for colorectal cancer diagnosis: a multicenter study
Source: Front Immunol. 2023 May 29;14:1126217. doi: 10.3389/fimmu.2023.1126217 (PMC10258350; doi:10.3389/fimmu.2023.1126217)
Supplement: Supplementary file 2 [file Image_2.pdf]

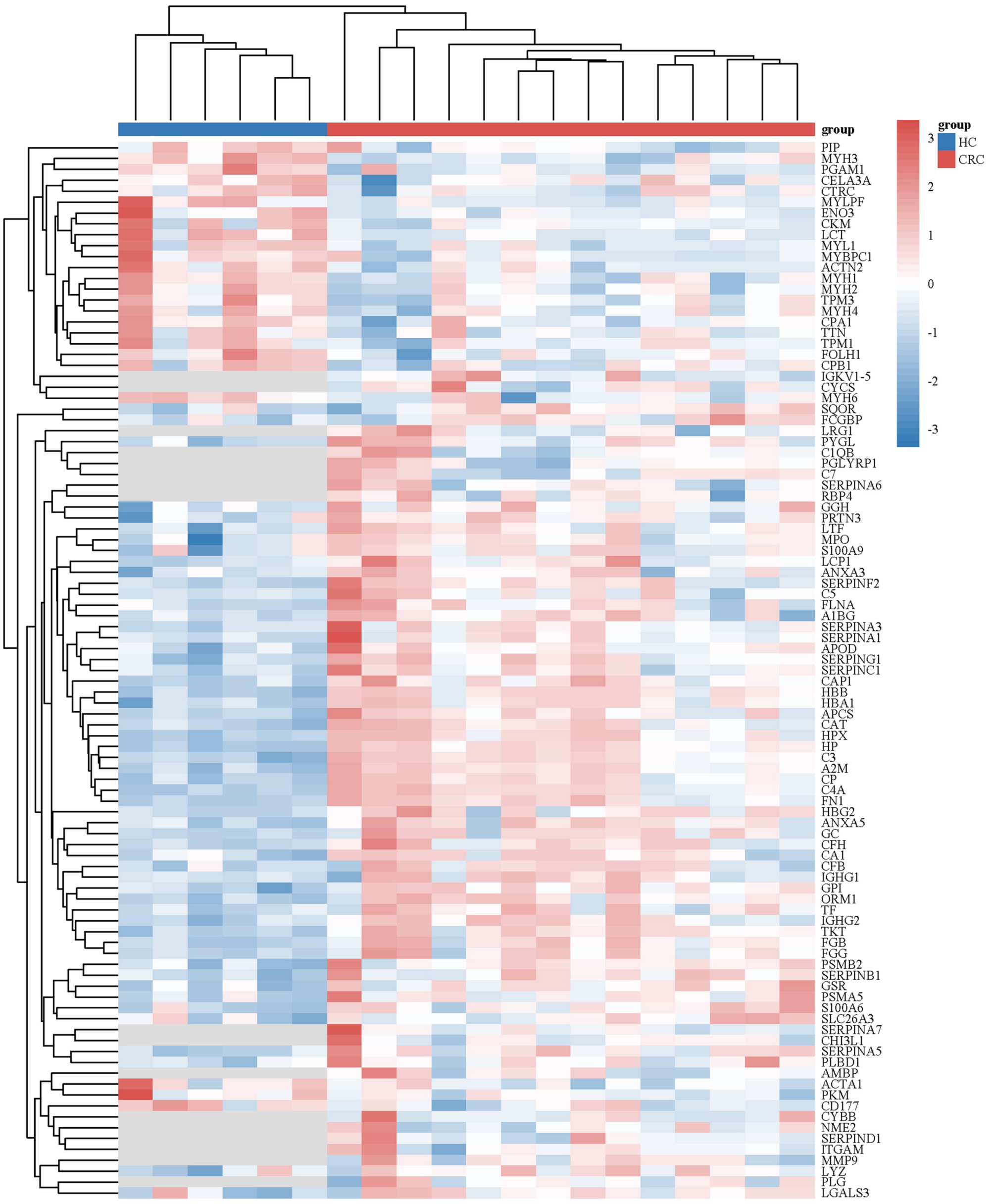

Supplementary Figure 2. Cluster heatmap of 97 differentially expressed fecal proteins. The criteria for differential expression of proteins were | $\log_2$-fold change | > 0.263 and $P < 0.05$.
