# Supplementary material for: Construction of a fecal immune-related protein-based biomarker panel for colorectal cancer diagnosis: a multicenter study
Source: Front Immunol. 2023 May 29;14:1126217. doi: 10.3389/fimmu.2023.1126217 (PMC10258350; doi:10.3389/fimmu.2023.1126217)
Supplement: Supplementary file 3 [file Image_3.pdf]

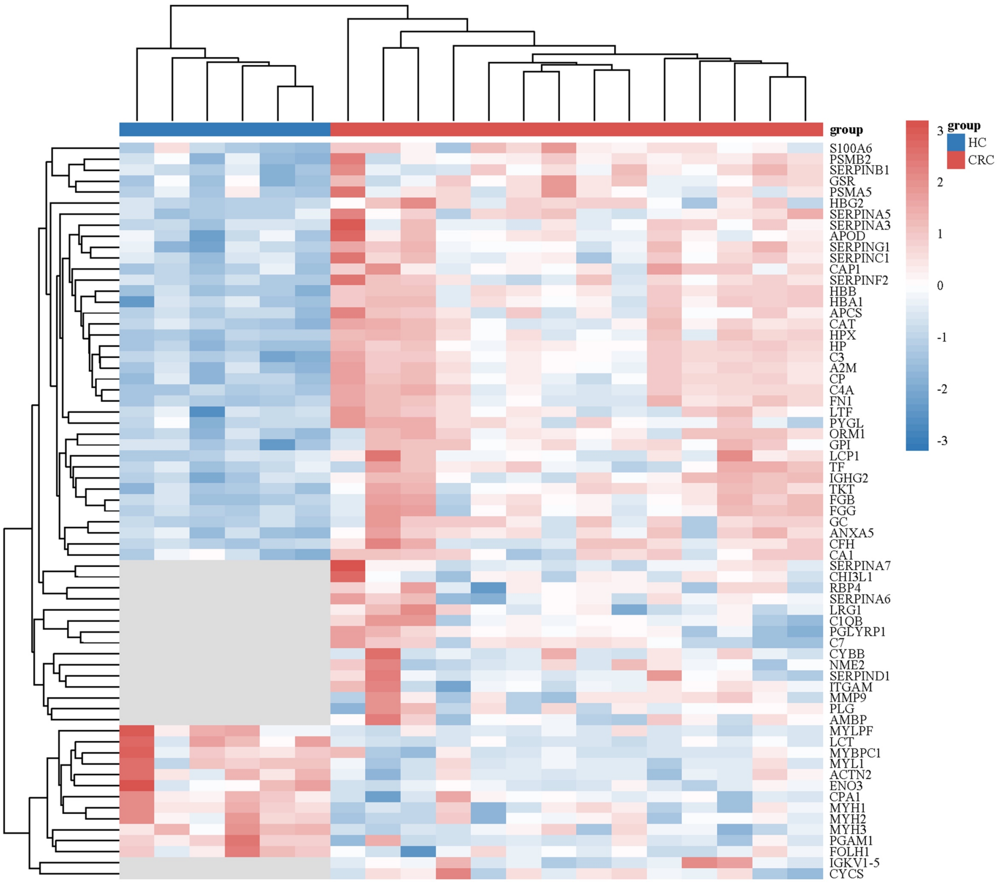

Supplementary Figure 3. Cluster heatmap of 67 differentially expressed stool proteins according to more stringent criteria. The criteria for differential expression of proteins were | $\log_2$-fold change | >1 and $P < 0.01$.
