# Supplementary material for: Construction of a fecal immune-related protein-based biomarker panel for colorectal cancer diagnosis: a multicenter study
Source: Front Immunol. 2023 May 29;14:1126217. doi: 10.3389/fimmu.2023.1126217 (PMC10258350; doi:10.3389/fimmu.2023.1126217)
Supplement: Supplementary file 4 [file Image_4.pdf]

A B

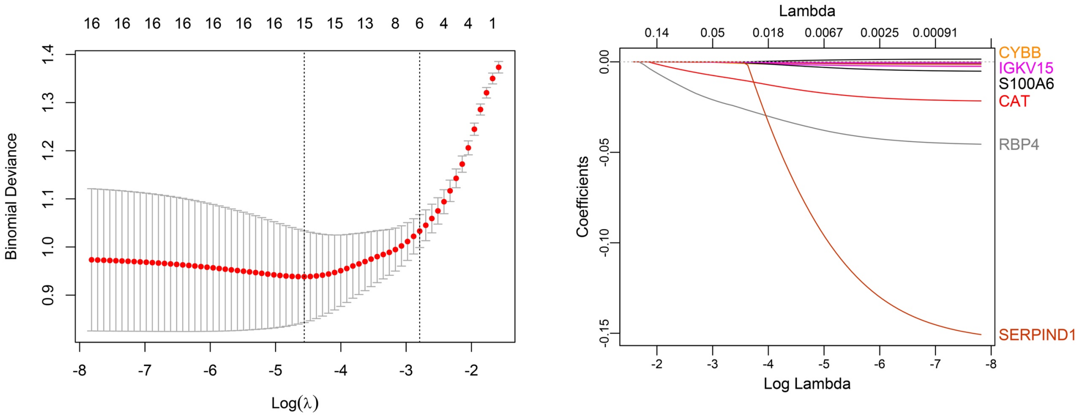

Supplementary Figure 4. Results for 16 fecal immune-related proteins in LASSO regression in the training set. (A) Feature selection using LASSO regularization. The y-axis represents the LASSO coefficient curve for the 16 proteins, and the bottom x-axis represents the logarithm of the penalty coefficient (lambda). The top x-axis represents the number of proteins. (B) Identification of the best penalty coefficient (lambda) for the LASSO model by 10-fold cross-validation based on the minimum criterion.
