# Supplementary material for: Construction of a fecal immune-related protein-based biomarker panel for colorectal cancer diagnosis: a multicenter study
Source: Front Immunol. 2023 May 29;14:1126217. doi: 10.3389/fimmu.2023.1126217 (PMC10258350; doi:10.3389/fimmu.2023.1126217)
Supplement: Supplementary file 5 [file Image_5.pdf]

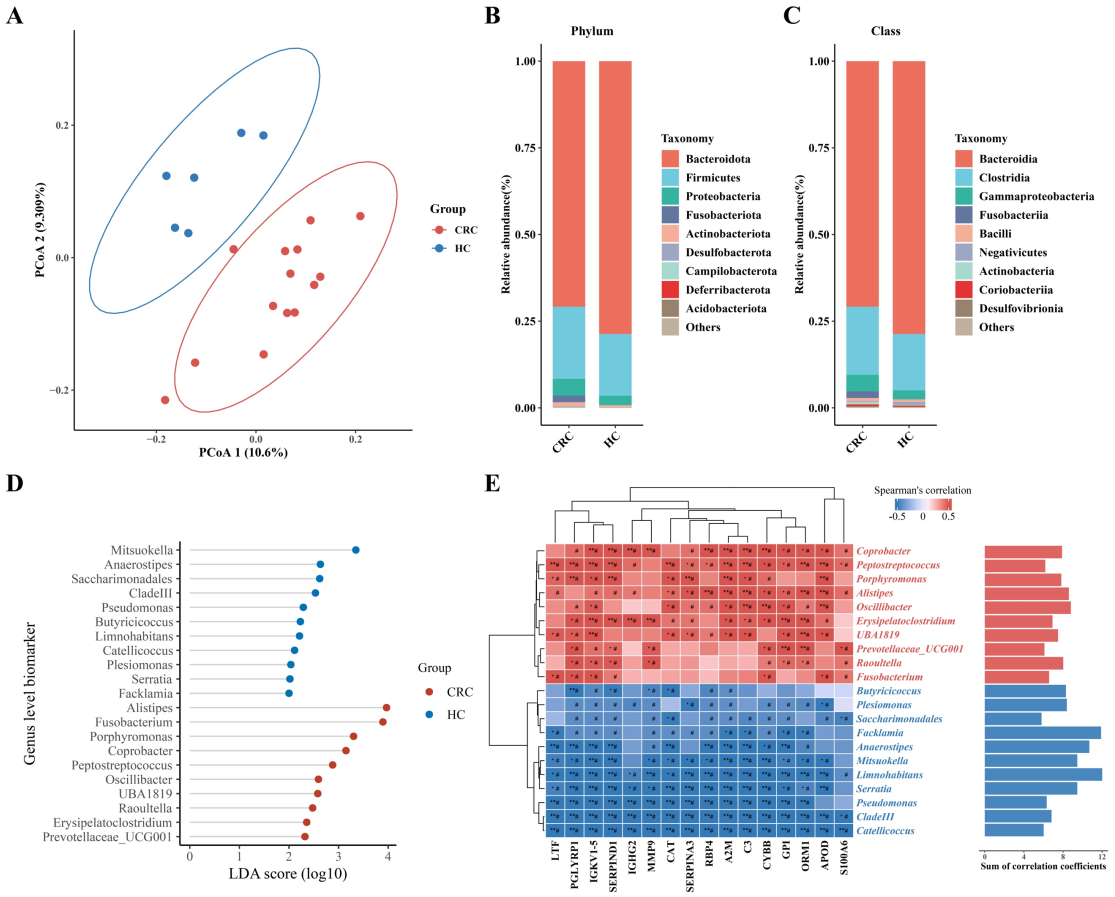

Supplementary Figure 5. Differentially abundant gut microbes between the CRC and HC groups. (A) PCoA plot based on the unweighted UniFrac distance of OTUs. Each point represents a sample. (B) Community histogram analysis showing the mean phylum relative abundance. (C) Community histogram analysis showing the mean class relative abundance. (D) The LDA score (genus level) based on LEfSe analysis identifies differentially abundant microorganisms that were enriched in CRC samples or HC samples. Blue dots represent microorganisms that were more highly enriched in HC samples, and red dots represent microorganisms that were more highly enriched in CRC samples. (E) Spearman correlation analysis of differentially abundant microorganisms (genus level) with 16 differential immune-related proteins in the discovery cohort. * represents the p value of the correlation analysis, $^{\#}$ represents the correlation coefficient, *, $P < 0.05$; **, $P < 0.01$; ***, $P < 0.001$; $^{\#}$, R > 0.4.
