# Supplementary material for: Construction of a fecal immune-related protein-based biomarker panel for colorectal cancer diagnosis: a multicenter study
Source: Front Immunol. 2023 May 29;14:1126217. doi: 10.3389/fimmu.2023.1126217 (PMC10258350; doi:10.3389/fimmu.2023.1126217)
Supplement: Supplementary file 6 [file Image_6.pdf]

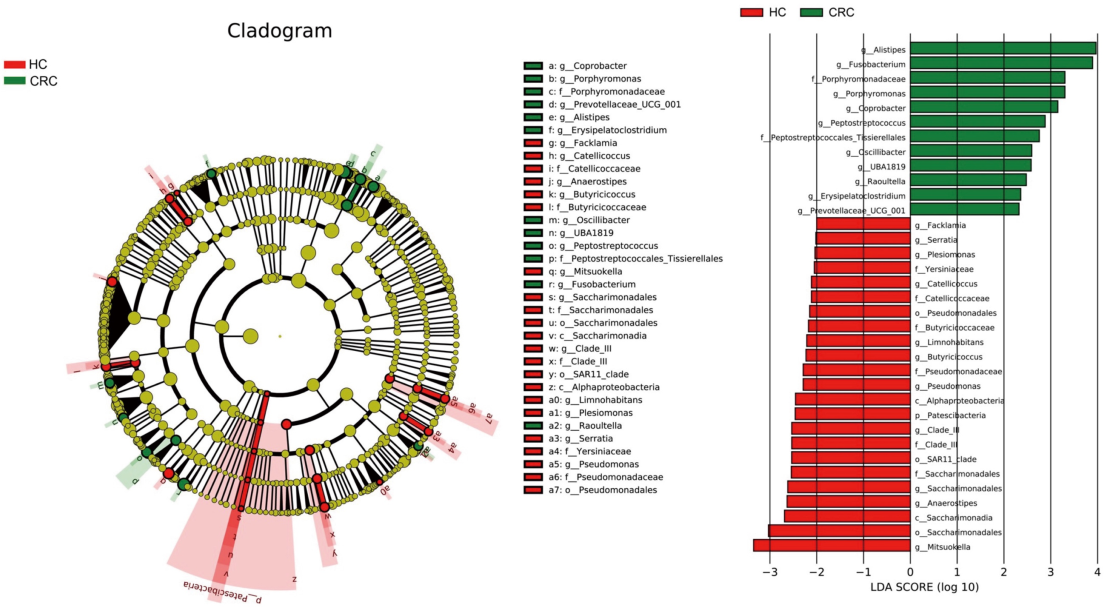

Supplementary Figure 6. Identification of differentially abundant microorganisms in the gut microbiota by LEfSe analysis. The criterion for identifying differentially abundant microbes is an absolute value of the LDA score > 2.
