# Supplementary material for: Construction of a fecal immune-related protein-based biomarker panel for colorectal cancer diagnosis: a multicenter study
Source: Front Immunol. 2023 May 29;14:1126217. doi: 10.3389/fimmu.2023.1126217 (PMC10258350; doi:10.3389/fimmu.2023.1126217)
Supplement: Supplementary file 7 [file Table_1.docx]

| **Supplementary Table 1.** Characteristics of population in this study. | | | | | | | | | |  | |  | |
| --- | --- | --- | --- | --- | --- | --- | --- | --- | --- | --- | --- | --- | --- |
| **Characteristic** | **Discovery cohort** | |  | **Validation cohort I** | |  | **Validation cohort II** | | $\text{χ}^{\text{2}}$ | | ***P* value** | |  |
|  | **n = 20** | |  | **n = 343** | |  | **n = 310** | |  |  |  |  |  |
| **Age, n (%)** |  |  |  |  |  |  |  |  | 4.134 | | 0.1266 | |  |
| ≥ 60(years) | 9 | (0.45) |  | 229 | (0.67) |  | 198 | (0.64) |  | |  | |  |
| < 60(years) | 11 | (0.55) |  | 114 | (0.33) |  | 112 | (0.36) |  | |  | |  |
| **Sex, n (%)** |  | (0) |  |  | (0) |  |  | (0) | 1.158 | | 0.5606 | |  |
| Male | 13 | (0.65) |  | 217 | (0.63) |  | 184 | (0.59) |  | |  | |  |
| Female | 7 | (0.35) |  | 126 | (0.37) |  | 126 | (0.41) |  | |  | |  |
| **Tumor depth, n (%)** | |  |  |  |  |  |  |  | 12.16 | | 0.1442 | |  |
| T1 | 0 | (0) |  | 22 | (0.11) |  | 25 | (0.18) |  | |  | |  |
| T2 | 6 | (0.43) |  | 32 | (0.17) |  | 26 | (0.18) |  | |  | |  |
| T3 | 7 | (0.50) |  | 93 | (0.48) |  | 59 | (0.42) |  | |  | |  |
| T4 | 1 | (0.07) |  | 27 | (0.14) |  | 17 | (0.12) |  | |  | |  |
| Unknown | 0 | (0) |  | 18 | (0.09) |  | 14 | (0.10) |  | |  | |  |
| **TNM stage, n (%)** | |  |  |  |  |  |  |  | 14.91 | | 0.061 | |  |
| I | 3 | (0.21) |  | 40 | (0.21) |  | 28 | (0.20) |  | |  | |  |
| II | 4 | (0.29) |  | 38 | (0.20) |  | 28 | (0.20) |  | |  | |  |
| III | 7 | (0.50) |  | 92 | (0.48) |  | 50 | (0.35) |  | |  | |  |
| IV | 0 | (0) |  | 4 | (0.02) |  | 6 | (0.04) |  | |  | |  |
| Unknown | 0 | (0) |  | 18 | (0.09) |  | 29 | (0.21) |  | |  | |  |
| CRC, Colorectal cancer; CRA, Colorectal adenoma; HC, Health control. Discovery cohort includes 14 CRC patients and 6 HCs. Validation cohort I includes 192 CRC patients and 151 HCs. Validation cohort II includes 141 CRC patients, 82 CRA patients, and 87 HCs. | | | | | | | | | | | | | |
