# Supplementary material for: Construction of a fecal immune-related protein-based biomarker panel for colorectal cancer diagnosis: a multicenter study
Source: Front Immunol. 2023 May 29;14:1126217. doi: 10.3389/fimmu.2023.1126217 (PMC10258350; doi:10.3389/fimmu.2023.1126217)
Supplement: Supplementary file 8 [file Table_2.docx]

| **Supplementary Table 2.** Sixteen immune-related proteins: Mean per sample with positive results, Fold change, and *P* value. | | | | | | | | | |
| --- | --- | --- | --- | --- | --- | --- | --- | --- | --- |
| **Gene name** | **Protein name** | **HC (n = 6)** | |  | **CRC (n =14)** | |  | **CRC vs. HC** | |
|  |  | **Mean per sample** | **Samples with positive results, n** |  | **Mean per sample** | **Samples with positive results, n** |  | **Fold change** | ***P* value** |
| A2M | Alpha-2-macroglobulin | 0.47 | 6 |  | 18.14 | 14 |  | 33.07 | < 0.001 |
| APOD | Apolipoprotein D | 1.72 | 6 |  | 5.23 | 14 |  | 2.71 | 0.002 |
| C3 | Complement C3 | 0.30 | 6 |  | 31.25 | 14 |  | 87.59 | < 0.001 |
| CAT | Catalase | 0.10 | 6 |  | 2.02 | 14 |  | 14.51 | < 0.001 |
| CYBB | Cytochrome b-245 heavy chain | 0.00 | 0 |  | 0.54 | 14 |  | ∞ | < 0.001 |
| GPI | Glucose-6-phosphate isomerase | 0.64 | 6 |  | 2.49 | 14 |  | 3.87 | < 0.001 |
| IGHG2 | Immunoglobulin heavy constant gamma 2 | 4.00 | 6 |  | 42.11 | 14 |  | 8.01 | < 0.001 |
| IGKV1-5 | Immunoglobulin kappa variable 1-5 | 0.00 | 0 |  | 1.46 | 14 |  | ∞ | < 0.001 |
| LTF | Lactotransferrin | 1.59 | 6 |  | 10.99 | 14 |  | 5.89 | 0.002 |
| MMP9 | Matrix metalloproteinase-9 | 0.00 | 0 |  | 0.48 | 14 |  | ∞ | < 0.001 |
| ORM1 | Alpha-1-acid glycoprotein 1 | 0.36 | 6 |  | 10.86 | 14 |  | 21.82 | < 0.001 |
| PGLYRP1 | Peptidoglycan recognition protein 1 | 0.00 | 0 |  | 0.52 | 14 |  | ∞ | < 0.001 |
| RBP4 | Retinol-binding protein 4 | 0.00 | 0 |  | 1.28 | 14 |  | ∞ | < 0.001 |
| S100A6 | S100 calcium-binding protein A6 | 1.49 | 6 |  | 3.10 | 14 |  | 2.11 | 0.002 |
| SERPINA3 | Alpha-1-antichymotrypsin | 6.21 | 6 |  | 21.43 | 14 |  | 2.51 | 0.008 |
| SERPIND1 | Heparin cofactor 2 | 0.00 | 0 |  | 0.69 | 14 |  | ∞ | < 0.001 |
