# Supplementary material for: Construction of a fecal immune-related protein-based biomarker panel for colorectal cancer diagnosis: a multicenter study
Source: Front Immunol. 2023 May 29;14:1126217. doi: 10.3389/fimmu.2023.1126217 (PMC10258350; doi:10.3389/fimmu.2023.1126217)
Supplement: Supplementary file 9 [file Table_3.docx]

| **Supplementary Table 3.** Multivariate logistic regression models predict CRC samples in the training set of Cohort II from six different hospitals in China. | | | |
| --- | --- | --- | --- |
| **Variables** | **Protein name** | **Odds ratio (95% CI)** | ***P*** |
| A2M, pg/g | Alpha-2-macroglobulin | 1.0000(1.0000, 1.0001) | 0.072 |
| CAT, pg/g | Catalase | 1.0162(1.0089, 1.0245) | < 0.001 |
| LTF, pg/g | Lactotransferrin | 1.0001(1.0001, 1.0002) | < 0.001 |
| MMP9, pg/g | Matrix metalloproteinase-9 | 1.0006(1.0003, 1.0010) | < 0.001 |
| RBP4, pg/g | Retinol-binding protein 4 | 1.0416(1.0182, 1.0690) | < 0.001 |
| SERPINA3, pg/g | Serpin A3 | 1.0009(1.0001, 1.0017) | 0.028 |
| CI, Confidence Interval. | | | |
