# Supplementary material for: Construction of a fecal immune-related protein-based biomarker panel for colorectal cancer diagnosis: a multicenter study
Source: Front Immunol. 2023 May 29;14:1126217. doi: 10.3389/fimmu.2023.1126217 (PMC10258350; doi:10.3389/fimmu.2023.1126217)
Supplement: Supplementary file 10 [file DataSheet_1.docx]

| **Supplementary File 1.** Product information for ELISA kits used in this study. | | | |
| --- | --- | --- | --- |
| **Gene name** | **Protein name** | **ELISA Manufacturer** | **Catalog Reference** |
| A2M | Alpha-2-macroglobulin | abcam | ab108888 |
| APOD | Apolipoprotein D | Cusabio | CSB-EL001935HU |
| C3 | Complement C3 | abcam | ab108823 |
| CAT | Catalase | abcam | ab277396 |
| CYBB | Cytochrome b-245 Beta Polypeptide | Finetest | EH4124 |
| GPI | Glucose-6-phosphate isomerase | biorbyt | orb406908 |
| IGHG2 | Immunoglobulin heavy constant gamma 2 | Abbexa | abx151962 |
| IGKV15 | Immunoglobulin kappa variable 1-5 | Abbexa | abx387966 |
| LTF | Lactotransferrin | abcam | ab200015 |
| MMP9 | Matrix metalloproteinase-9 | abcam | ab246539 |
| ORM1 | Alpha-1-acid glycoprotein 1 | abcam | ab243675 |
| PGLYRP1 | Peptidoglycan recognition protein 1 | biorbyt | orb441539 |
| RBP4 | Retinol-binding protein 4 | abcam | ab196264 |
| S100A6 | Protein S100-A6 | Biomatik | EKE60801 |
| SERPIND1 | Serpin family D member 1 | abcam | ab277402 |
| SERPINA3 | Alpha-1-antichymotrypsin | abcam | ab157706 |
|  | Hemoglobin | abcam | ab157707 |
