# Supplementary material for: Construction of a fecal immune-related protein-based biomarker panel for colorectal cancer diagnosis: a multicenter study
Source: Front Immunol. 2023 May 29;14:1126217. doi: 10.3389/fimmu.2023.1126217 (PMC10258350; doi:10.3389/fimmu.2023.1126217)
Supplement: Supplementary file 11 [file DataSheet_2.docx]

Supplementary manuscript

1. MATERIALS AND METHODS

1.1 16S rRNA sequencing

Genomic DNA of the samples was extracted using DNA extraction kits, followed by DNA concentration measurement using agarose gel electrophoresis and a NanoDrop2000. PCR amplification of the V3-V4 hypervariable regions of the bacterial 16S rRNA gene was carried out in a 25 μl reaction using universal primer pairs (343F: 5′-TACGGRAGGCAGCAG-3′; 798R: 5′-AGGGTATCTAATCCT-3′). Specific barcoded primers and Takara's Tks Gflex DNA Polymerase were used to ensure amplification efficiency and accuracy. The PCR products were detected using electrophoresis, purified using magnetic beads after detection, purified as a second-round PCR template, subjected to second-round PCR amplification, detected again using electrophoresis, purified using magnetic beads after detection, and quantified with a Qubit fluorometer after purification. Aliquots were mixed according to PCR product concentrations, followed by sequencing. The raw sequencing data were in FASTQ format.

The raw paired-end sequences were dehybridized using Trimmomatic software version 0.35(1). The dehybridized paired-end sequences were spliced using FLASH software version 1.2.11(2). Sequences containing ambiguous bases, single-base high-repeat regions (homologous), and sequences that were too short were removed again using split libraries in QIIME(3). Additionally, chimeric sequences were detected and removed from the sequences using UCHIME. After preprocessing the sequencing data to generate high-quality sequences, OTU classification was performed using Vsearch software version 2.4.2(4) at 97% similarity. Representative sequences for each OTU were selected using the QIIME software package, and all representative sequences were annotated against the database. The 16S sequences were compared using the Silva database, and species comparison annotation was performed using RDP classifier software(5); annotation results with confidence intervals greater than 0.7 were retained. Differentially abundant microorganisms between the two groups were determined using linear discriminant analysis (LDA) effect sizes (LEfSe) with absolute values of LDA scores >2.

2. RESULTS

2.1 Fecal immune-related proteins were associated with a disordered gut microbiota in CRC

Disorder of the gut microbiota affects gut immune activity and contributes to CRC progression(6), which may also account for the changes in immune-related proteins in in the stool samples from CRC patients. Subsequently, 16S rRNA sequencing was performed on stool samples in the discovery cohort. Principal coordinate analysis (PCoA) based on unweighted UniFrac distances showed differences between samples in the CRC and HC groups (Supplementary Figure 5A). Analysis of the species composition at the phylum level (Supplementary Figure 5B) and class level (Supplementary Figure 5C) also showed differences in microbial composition between the CRC and HC groups. Differences in the microbial compositions between the CRC and HC groups were identified based on LEfSe, where the absolute value of the LDA score was greater than 2 (Supplementary Figure 6). The results showed that 10 microbial genera were enriched in the CRC group, including *Alistipes* and *Fusobacterium* (Supplementary Figure 5D). The other 11 microbes were more enriched in the HC group stool samples. The results of Spearman correlation analysis showed that 16 immune-associated proteins were positively correlated with the CRC-enriched microbes and negatively correlated with HC-enriched microbes (Supplementary Figure 5E).

The above results indicated that the altered immune-related proteins in stool may be associated with the gut microbes with altered abundances in CRC patients.

****References****

1. Bolger AM, Lohse M, Usadel B. Trimmomatic: a flexible trimmer for Illumina sequence data. *Bioinforma Oxf Engl* (2014) 30:2114–2120. doi: 10.1093/bioinformatics/btu170

2. Reyon D, Tsai SQ, Khayter C, Foden JA, Sander JD, Joung JK. FLASH assembly of TALENs for high-throughput genome editing. *Nat Biotechnol* (2012) 30:460–465. doi: 10.1038/nbt.2170

3. Caporaso JG, Kuczynski J, Stombaugh J, Bittinger K, Bushman FD, Costello EK, Fierer N, Peña AG, Goodrich JK, Gordon JI, et al. QIIME allows analysis of high-throughput community sequencing data. *Nat Methods* (2010) 7:335–336. doi: 10.1038/nmeth.f.303

4. Rognes T, Flouri T, Nichols B, Quince C, Mahé F. VSEARCH: a versatile open source tool for metagenomics. *PeerJ* (2016) 4:e2584. doi: 10.7717/peerj.2584

5. Wang Q, Garrity GM, Tiedje JM, Cole JR. Naive Bayesian classifier for rapid assignment of rRNA sequences into the new bacterial taxonomy. *Appl Environ Microbiol* (2007) 73:5261–5267. doi: 10.1128/AEM.00062-07

6. Okumura S, Konishi Y, Narukawa M, Sugiura Y, Yoshimoto S, Arai Y, Sato S, Yoshida Y, Tsuji S, Uemura K, et al. Gut bacteria identified in colorectal cancer patients promote tumourigenesis via butyrate secretion. *Nat Commun* (2021) 12:5674. doi: 10.1038/s41467-021-25965-x
